# Controlled release of ciprofloxacin and ceftriaxone from a single ototopical administration of antibiotic-loaded polymer microspheres and thermoresponsive gel

**DOI:** 10.1371/journal.pone.0240535

**Published:** 2020-10-12

**Authors:** Liza A. Bruk, Katherine E. Dunkelberger, Pawjai Khampang, Wenzhou Hong, Srivatsun Sadagopan, Cuneyt M. Alper, Morgan V. Fedorchak

**Affiliations:** 1 Department of Bioengineering, University of Pittsburgh, Pittsburgh, PA, United States of America; 2 Department of Otolaryngology and Communication Sciences, Medical College of Wisconsin, Milwaukee, WI, United States of America; 3 Department of Neurobiology, University of Pittsburgh, Pittsburgh, PA, United States of America; 4 Department of Otolaryngology, University of Pittsburgh, Pittsburgh, PA, United States of America; 5 Division of Pediatric Otolaryngology, UPMC Children’s Hospital of Pittsburgh, Pittsburgh, PA, United States of America; 6 Clinical and Translational Science Institute, University of Pittsburgh, Pittsburgh, PA, United States of America; 7 Department of Ophthalmology, University of Pittsburgh, Pittsburgh, PA, United States of America; 8 Department of Chemical Engineering, University of Pittsburgh, Pittsburgh, PA, United States of America; Universidade Federal de Sao Paulo/Escola Paulista de Medicina (Unifesp/epm), BRAZIL

## Abstract

Acute otitis media (AOM) is the main indication for pediatric antibiotic prescriptions, accounting for 25% of prescriptions. While the use of topical drops can minimize the administered dose of antibiotic and adverse systemic effects compared to oral antibiotics, their use has limitations, partially due to low patient compliance, high dosing frequency, and difficulty of administration. Lack of proper treatment can lead to development of chronic OM, which may require invasive interventions. Previous studies have shown that gel-based drug delivery to the ear is possible with intratympanic injection or chemical permeation enhancers (CPEs). However, many patients are reluctant to accept invasive treatments and CPEs have demonstrated toxicity to the tympanic membrane (TM). We developed a novel method of delivering therapeutics to the TM and middle ear using a topical, thermoresponsive gel depot containing antibiotic-loaded poly(lactic-co-glycolic acid) microspheres. Our *in vitro* and *ex vivo* results suggest that the sustained presentation can safely allow therapeutically relevant drug concentrations to penetrate the TM to the middle ear for up to 14 days. Animal results indicate sufficient antibiotic released for treatment from topical administration 24h after bacterial inoculation. However, animals treated 72h after inoculation, a more clinically relevant treatment practice, displayed spontaneous clearance of infection as is also often observed in the clinic. Despite this variability in the disease model, data suggest the system can safely treat bacterial infection, with future studies necessary to optimize microsphere formulations for scaled up dosage of antibiotic as well as further investigation of the influence of spontaneous bacterial clearance and of biofilm formation on effectiveness of treatment. To our knowledge, this study represents the first truly topical drug delivery system to the middle ear without the use of CPEs.

## 1. Introduction

### 1.1. Incidence and severity of acute otitis media

Acute otitis media (AOM), characterized by middle ear inflammation, acute onset, and duration of illness ranging from several days to two weeks, accounts for 20 million pediatrician visits annually and is the main indication for pediatric antibiotic prescription in the United States [1–4]. In fact, over 25% of antibiotic prescription is for the sole purpose of treating AOM [2,3]. Approximately 80% of children under three years old experience AOM at least once [2,3] and 30–40% of children experience at least six instances of AOM before the age of seven [5,6]. Recurrent AOM can develop into chronic otitis media, which often requires surgical intervention such as myringotomy and placement of tympanostomy tubes for ventilation and drainage [7–9]. Chronic otitis media has also been associated with sensorineural hearing loss, which may lead to speech and learning deficits [9–13]. In the US alone, 500,000 children undergo tympanostomy surgery annually at a cost of $5 billion [14]. Developing countries see over 10 times more incidence of OM than the US and European nations [7,10,15].

### 1.2. Current clinical standards and associated shortcomings

Oral antibiotics remain the standard of care, although such treatment is recognized as impractical due to the risks of systemic side effects and antibiotic resistance associated with persistent biofilm formation [3,4,16–18]. Further, oral antibiotics may not provide adequate drug concentration to the middle ear to clear these persistent biofilms [19,20]. Though less frequently prescribed for AOM, and only in cases of non-intact TM due to perforation or placement of ventilation tubes, topical ear drops are also problematic, with less than 10% of the applied drug reaching the middle ear [21]. For example, 0.2% ciprofloxacin is, if applied correctly to non-intact TM, delivered in quantities of 1mg/day [22]. The minimum inhibitory concentration (MIC) of ciprofloxacin to inhibit microorganisms commonly responsible for OM, including *Streptococcus pneumoniae* and *Haemophilus influenzae* [18], has been determined to be up to 1–2μg/mL [23,24]. Thus with proper instillation, the standard topical antibiotic drop treatment [22] can deliver up to 1000 times excess antibiotic to the middle ear and surrounding tissues, which can contribute to local and systemic side effects much like their oral counterparts while not always effectively clearing infection due to limitations including low permeability and antibiotic resistant infections [25]. Further limitations of standard topical ear drops include low patient compliance, particularly when self-medicating and for treatment durations greater than 1 week, associated with high dosing frequency and difficulty of administration [26–28]. An ideal controlled release system should be simple to use and deliver to the middle ear an antibiotic dose closer to the target MIC at a frequency that is manageable by patients and/or their caregivers.

### 1.3. Pipeline products

Accordingly, research has focused on expanding and improving topical antibiotic treatment to address the aforementioned shortcomings. Studies by Otonomy, Inc. have shown success in releasing drugs from hydrogel vehicles with sustained release and efficacy in clearing infection and inflammation observed from 2 weeks to 3 months, but these systems require invasive procedures through placement either via intratympanic injection or during tympanostomy surgery [29–31]. Further, the gel cannot be retrieved from the middle ear if there are complications and long-term toxicity effects due to degradation byproducts have not been investigated.

Topical sustained release systems have also been investigated to improve AOM treatment in cases with intact TMs, including ciprofloxacin-loaded hydrogels augmented by combinations of chemical permeation enhancers (CPEs), which resulted in increased transtympanic permeation [21,32,33]. CPEs are useful for increasing permeability through the stratum corneum, however inherent inflammation due to OM may increase permeability of the eardrum to sustained presentation of topical antibiotics without the use of CPEs [9,21]. Further, CPEs carry a risk of toxic side effects [34] and have been shown to be cytotoxic during the preclinical validation of this system, with only 20% keratinocyte viability after 3 days [32]. Additionally, recent studies suggest that outcomes may be improved when antibiotic treatment regimens lasts 10 or more days [35], currently unachievable by these hydrogel-only systems. Achieving this duration of drug release typically requires a secondary controlled release vehicle such as hydrolytically degradable polymer microspheres [36]. Degradable microspheres offer the ability to sustain delivery of drug for an extended period of time from a single, localized dose, tunability for various drugs and dosing ranges, and consistent daily release of therapeutic concentrations [21].

Ciprofloxacin is an ideal candidate for encapsulation and has been extensively utilized in the aforementioned studies due to local toxicity effects of other standard topical antibiotics including tobramycin, neomycin, and gentamycin [16,37], effects which have not been observed due to ciprofloxacin treatment [38]. Further, ciprofloxacin is a fluoroquinolone that affects most of the microorganisms responsible for OM, and although it is a rarely occurring pathogen in AOM, ciprofloxacin is in fact the only antibiotic that treats *Pseudomonas*-based OM [16,38]. Additionally, several of the bacterial strains most common in OM, including *Haemophilus influenzae* and *Streptococcus pneumoniae* readily form biofilms, which may inhibit the effectiveness of antibiotic therapy [17,18]. Although less commonly used than other treatments, the current treatment when there is suspicion of resistance or low patient compliance is a three-day course of intramuscular ceftriaxone treatment, which has shown to be as effective as a two-week treatment course of amoxicillin and has proven effective in treatment of penicillin-resistant infections and persistent *Streptococcus*-based infections [39–43]. Therefore, to maximize efficacy of treatment, ciprofloxacin and ceftriaxone were chosen for encapsulation in the drug delivery system described herein.

There is a clear need for safe, simple, and effective drug delivery to the ear for treatment of otitis media. The goal of the studies described herein was to develop and test an innovative method of sustained presentation of antibiotics to and across the TM. Previous work by this group has shown that ocular permeability barriers can be overcome using a validated drug delivery system including a topical, thermoresponsive gel depot containing drug-loaded poly(lactic-co-glycolic acid) (PLGA) microspheres (MS) [44,45]. Although there are similarities in these topical routes of administration which indicated the potential for the system to be effective as an otic topical drug delivery system, the drug presentation to the ear anatomy is quite different from ocular presentation and lends to novelty of the system. Therefore, sustained presentation to inflamed TM afforded by the stable gel depot is proposed to allow a therapeutically relevant amount of drug to penetrate the TM without harmful additives. We hypothesized that development and optimization of a novel drug delivery system can noninvasively sustain localized antibiotic release to the TM and middle ear, as demonstrated by *in vitro* and *in vivo* testing.

## 2. Materials and methods

### 2.1. Microsphere fabrication and characterization

All materials were obtained from Sigma-Aldrich (St Louis, MO) unless otherwise noted. Ciprofloxacin-loaded microspheres were prepared using a water-in-oil-in-water (W/O/W) double emulsion procedure adapted from our previous work [46–48]. In brief, 200mg poly(lactic-co-glycolic) acid (PLGA) (MW 24–38 kDa; viscosity 0.32–0.44dL/g) were dissolved in 4mL dichloromethane to which 250μL of 100mg/mL ciprofloxacin in 1M acetic acid was added. The dissolved drug and polymer mixture were then sonicated for 10 seconds at 30% amplitude (EpiShear Probe Sonicator, Active Motif, Carlsbad, CA) followed by homogenization in 60mL of 2% poly(vinyl alcohol) (PVA) (Polysciences, Warrington, PA) for 1 minute at 7000rpm (Silverson L5M-A, East Longmeadow, MA). The resulting liquid-phase emulsion was added to 80mL of 1% PVA and stirred at 600rpm for 3 hours, resulting in precipitation of solid microspheres (MS). Drug-loaded and blank MS, fabricated by substituting deionized (DI) water for aqueous drug, were then washed 4 times by centrifugation, resuspended in DI water, flash frozen in liquid nitrogen, and lyophilized for 48–72 hours (Speedvac Freezone, Labconco, Kansas City, MO). Ceftriaxone-loaded MS were prepared via an oil-in-water (O/W) single emulsion procedure in which 200μL of a 30mg/mL solution of ceftriaxone in dimethyl sulfoxide (DMSO) was added to dissolved polymer and followed by homogenization at 3000rpm and subsequent steps as described above, with 432.5mg NaCl added to 1% PVA and 305.8mg NaCl added to 2% PVA to balance the osmolality of the ceftriaxone solution.

Size, shape, porosity, and drug release were characterized for each set of MS prepared. Scanning electron microscopy (SEM) was used to examine shape and morphology of both blank and drug-loaded MS (JEOL JSM 6335F, Peabody, MA). MS diameter was quantified by volume impedance measurements with mean and standard deviation determined for approximately n = 15,000 MS per sample (Multisizer, Beckman Coulter, Brea, CA). Density of MS was determined via a measurement device custom-made using a 1mL syringe. A known mass, approximately 15-30mg, of MS was added and MS packed by pushing down on the plunger, allowing for volume to be determined and density calculated from the known mass and volume.

*In vitro* drug release kinetics were determined using 10mg of MS suspended in phosphate buffered saline (PBS) and continuously rotated at 37°C. The supernatant was removed via centrifugation every 24 hours and replaced with fresh PBS. Ciprofloxacin concentration in the supernatant was quantified via UV/Vis absorbance measures taken at 246nm (SoftMax Pro 5, Molecular Devices, Sunnyvale, CA), with background signal from blank MS subtracted from each measurement and regressed against the standard curve, validated for 1–10μg/ml. High performance liquid chromatography (HPLC; 1220 Infinity LC, Agilent Technologies, Santa Clara, CA) using the following settings was used to confirm: Kromasil C18 column (4.6mm x 150mm, 3.5μm particles), 10μL injection volume, 80:20 acetonitrile:0.1% trifluoracetic acid mobile phase, 1mL/min flow rate, detection at 275nm. This method has been reported previously [21] and validated by our group for the range 100ng/mL-10mg/mL. Ceftriaxone concentrations were quantified using the following settings adapted from previously reported studies [49]: reverse phase Zorbax Eclipse Plus C18 Column (4.6mm × 150 mm, 5μm particles), 20μL injection volume, 70:5:25 HPLC water:acetic acid:acetonitrile mobile phase, flow rate 1mL/min, detection at 254nm. For each formulation, total drug loading was taken to be cumulative drug release when all MS were fully degraded, and therefore drug release was exhausted, after 14 days.

### 2.2. Thermoresponsive gel fabrication, characterization, and retention

The gel was prepared via free radical polymerization of N-isopropylacrylamide (NIPAAm) (Fisher Scientific, Waltham, MA) and poly(ethylene glycol) (MW ~200kDa), in the presence of ammonium persulfate (APS) and tetramethylethylenediamine (TEMED). The gel precursor was refrigerated for 24h and then washed in DI water at ~40°C. The gel formulation underwent testing for degradation rate, swelling ratio, and lower critical solution temperature (LCST) as previously reported in Fedorchak et al 2017 [48]. Briefly, LCST, the temperature at which gelation occurs [50], was determined via absorbance measurements at 415nm over a temperature range of 25–40°C, increased by 1°C increments and allowing 15 minutes between readings for temperature equilibration. Degradation rate was determined by comparing mass of liquid gel at baseline and every 7 days for 28 days in the gel phase, with the gel stored in PBS at 37°C. Swelling ratio was determined by placing 100μL of gel in 37°C for 72h or until the liquid was fully evaporated. Dry mass was compared to swollen mass prior to evaporation for n = 3 samples.

To qualitatively analyze retention of gel in the ear canal, a transparent plastic human ear model was used. A 300μg/mL solution of FITC in DI water was prepared and 50μL of the solution was added and mixed with 200μL of gel. Using a 1mL syringe with capillary tubing attached to the tip, 100μL of fluorescently dyed gel was applied to the ear canal on the tympanic membrane (TM). The model was placed in a 100mL beaker with the ear canal facing up and left in a 37°C incubator for 30 minutes for the gel to solidify. After 30 minutes, the ear model was removed from the beaker and placed in the incubator with the ear canal perpendicular to the bottom of the incubator. The ear model was evaluated by visual inspection and photography after 2, 3, and 24 hours.

### 2.3. Cytotoxicity

Cytotoxicity of the MS was analyzed by both Vybrant MTT (3-(4,5-dimethylthiazol-2yl)-2,5-diphenyltetrazolium bromide) Cell Proliferation Assay Kit and LIVE/DEAD Viability/Cytotoxicity Kit (Thermo Fisher Scientific, Waltham, MA). Studies were carried out using human primary epidermal keratinocytes (ATCC, Manassas, VA), the primary cell type found in TM tissue [51]. In brief, ~3,000 cells/well were plated in 96 well plates and incubated in 200μL Dermal Cell Basal Medium with Keratinocyte Growth Kit (ATCC) for 24h at 37°C with 5% CO_2_ to achieve a monolayer. Cells with medium only were used as the positive control for viability in both assays and cells incubated with 70% methanol for 5-10mins prior to each assay were used as a negative control, per kit instructions. All test groups included 100μL medium and 100μL test material: PBS, 1μg/mL ciprofloxacin (CIP), 5μg/mL ceftriaxone (CFX), 1mg blank MS, 1mg CIP MS, 1mg CFX MS, blank MS releasate, CIP MS releasate, CFX MS releasate. Releasates were collected via the same methods as described above, with only 24h releasates used for this study as highest release for both antibiotics is seen after 24h: 0.79 ± 0.07μg ciprofloxacin and 1.58 ± 0.03μg ceftriaxone. Once a monolayer of cells was achieved, treatment groups were applied and incubated for an additional 24h. Microspheres were also applied to wells with no cells to account for any background signal. MTT assay was performed by additional incubation for 4h with 10μL MTT stock solution, followed by incubation for 10mins with 50μL dimethylsulfoxide (DMSO). Absorbance in each well was then determined via spectrophotometer at 540nm. Percent viability was determined by normalizing to 100% viability in the positive control group.

For the LIVE/DEAD assay, the same test groups were used as above. The assay was performed via addition of 100μL LIVE/DEAD working solution (2μm calcein AM/4μm ethidium homodimer-1) to each well and incubating for 45 minutes at 25°C prior to measuring absorbance at 530nm and 645nm to detect live and dead cells, respectively. Percent viability was determined by normalizing to 100% viability in the positive control group. The mean and standard deviation absorbance values for both assays were determined for n = 3 samples in each test group. Gel cytotoxicity has been previously investigated and reported by this group in Fedorchak et al 2017 [48].

### 2.4. Ex vivo transtympanic permeation

Dunkin-Hartley guinea pigs (GPs) of both sexes were purchased from Charles River. All animal studies were performed with approval from and in accordance with the University of Pittsburgh Institutional Animal Care and Use Committee (IACUC) standards. Forty-four GPs were used for this study and randomized into three groups: 21 in the negative control group (100μL gel with 10mg blank MS), 21 in the test group (100μL with 10mg ciprofloxacin-loaded MS), and 2 in the positive control group (0.2% weight/volume ciprofloxacin).

Animals were humanely sacrificed via anesthesia with isoflurane followed by an overdose of intracardiac sodium pentobarbital. Ear canals and tympanic membranes were harvested according to guidance by an otolaryngology lab technician. In brief, after removal of the head and lower jaw, the skull was bisected in a sagittal cut, allowing for visualization of the bullae. The bulla and external ear canal were extracted from either side using surgical scissors and as much tissue as possible was removed using scalpel and forceps. The bullae were carefully bisected via transverse cut using surgical scissors to expose the TMs without damaging their integrity. The TMs were visually inspected using a dissecting microscope to confirm lack of perforation. The ear canals were suspended in 10mL PBS in 50mL beakers with the TM parallel to the bottom of the beaker and only the TM surface in contact with the PBS. Due to *ex vivo* tissue degradation, it was necessary to use 3 freshly harvested ears daily for the 14-day study duration.

For the negative control and test groups, 100μL gel mixed with 10mg of MS (blank and ciprofloxacin-loaded, respectively) was applied to the TM. Microspheres were aged for days 1–14 (in triplicate) by suspending in PBS and rotating in a 37°C incubator. Each day, 3 aliquots of MS were centrifuged, supernatant removed, and MS resuspended in gel. MS were suspended in gel by pipetting gel onto pre-weighed MS and mixing with pipette tip or needle. Topical administration from a standard 0.2% ciprofloxacin drop is applied daily in the same amount, resulting in equal drug concentration delivered daily [22]. Therefore, only one time point with 3 ears was observed for the positive control group and transtympanic concentration was assumed to be equal for each day. These drops were instilled in the same volume as in humans (250μL) [22], however due to physiological differences in ear canal volume between guinea pigs and humans [52–55], resulting drug concentration in receiving chamber was scaled down accordingly for comparison.

To determine drug concentration in the PBS receiving chamber, 1mL was removed and frozen daily and all 10mL in the beaker replaced with fresh PBS. All samples were centrifuged at 3500rpm for 5 minutes, then 500μL of supernatant from each was removed and centrifuged in 10kDa filters (Amicon Ultra, Merck Millipore, Cork, Ireland) at 8200rpm for 10 minutes. Drug concentration was determined both by spectrophotometry and HPLC analysis as previously described.

### 2.5. Bacterial killing studies

Non-typeable *Haemophilus influenzae* (strain 86-028NP) bacteria were cultured at a concentration of approximately 10^6^ CFU/mL. To these bacterial cultures, treatment groups of blank MS, ciprofloxacin-loaded MS, ceftriaxone-loaded MS, 1μg/mL ciprofloxacin, 5μg/mL ceftriaxone, or no treatment were applied. All MS groups were added in a concentration of 10mg MS/mL bacterial culture medium to approximate intended treatment dose of 10mg MS, containing 2.56 ± 0.08μg ciprofloxacin or 2.71 ± 0.02μg ceftriaxone. Concentrations of free antibiotic for positive controls were chosen based on literature values for minimum inhibitory concentrations of ciprofloxacin and ceftriaxone to *H*. *influenzae* and other relevant bacteria [23,24] and were confirmed by this group. After incubation for 24–48 hours at 37°C, viable bacteria were counted by serially diluting the cultures followed by plating on agar plates and incubating at 37°C for 24–48 hours.

### 2.6. Auditory brainstem responses

Animal studies conformed to the NIH Guide for Care and Use of Laboratory Animals and all procedures were approved by the University of Pittsburgh IACUC. Two groups of n = 3 guinea pigs were used to test the effect of the administration of the gel system on conductive hearing sensitivity using the auditory brainstem response (ABR) with free-field sound presentation. Each animal underwent ABR testing under the following conditions: both ears unplugged, left ear plugged with foam and dental silicone, left ear plugged and gel applied to the right ear, gel in right ear after removing plug from left ear, both ears unplugged after removal of gel. To plug the left ear, a piece of foam was inserted into the ear canal, covered with vinyl polysiloxane impression material (Examix NDS, GC America, Alsip, IL), and allowed to set for 5 mins. Gel was instilled in volumes of 25 or 100 μL to the right ear using a 200μL pipette and allowed to set for 5 mins. Gelation and presence of gel in the ear canal were confirmed via visual inspection using an otoscope.

ABR experiments were performed under 1–2% isoflurane anesthesia, with ABR acquired via three subcutaneous electrodes (27-gauge needle electrodes, Rochester Electro-medical) placed at standard locations (signal–vertex of skull, reference–below pinna overlying mastoid, ground–below the other pinna). Click stimuli (100μs long) with alternating polarity repeating every 100ms (10 per second) were presented ~1000 times each at 14 sound levels (5dB-75dB in 5dB steps), with the loudest sound level presented first (total duration ~27min). ABR signals were collected at 30kHz sampling rate and bandpass filtered (200-2000Hz). A rejection criterion of peak-to-peak response greater than 50μV was used to discard trials that included potential artifacts. The distributions of baseline fluctuations at each sound level was obtained from a 2.5-ms segment of the ABR recording just prior to the onset of each click. Wave 1 of the ABR at the loudest stimulus level was defined as the first positive peak after stimulus onset. At the other sound levels, wave 1 was defined as the first positive peak occurring at a time equal to or later than wave 1 at the loudest level. The peak-to-peak amplitude (wave 1 trough to wave 2 peak) was taken as the ABR amplitude on each trial. The hearing threshold of the tested ear was taken to be the lowest sound level at which the mean ABR amplitude exceeded 4 standard deviations of the distribution of baseline fluctuations.

### 2.7. In vivo safety and efficacy studies

Chinchillas of both sexes were purchased from Moulton Chinchilla Ranch (Rochester, Minnesota) and all studies were performed with approval from and in accordance with the Medical College of Wisconsin IACUC standards. All studies were performed humanely under anesthesia and bupivacaine was administered prophylactically to prevent any potential pain associated with bacterial infection and subsequent treatment. The animals were randomized into four groups: no treatment control, treatment with blank MS/gel, treatment with ciprofloxacin MS/gel, treatment with ceftriaxone MS/gel. For MS/gel groups, MS were mixed with gel as previously described, by pipetting gel onto the MS and mixing with pipette tip or needle. One cohort of animals were inoculated with 1.94 x10^5^ CFU/ear non-typeable *Haemophilus influenzae* (strain 86-028NP) via transbullar injection and received treatment after 24h, with the contralateral ear as internal control. For subsequent studies, all animals were inoculated and treated in both ears 72h after inoculation. Previous studies by other groups have also treated animals 72h after inoculation [32,33]. After infection was allowed to develop, tympanic membranes were imaged with MedRx otoscope (Largo, FL) and gel/MS was instilled to treatment groups using a 1mL syringe with 18G needle. At time points including days 1, 3, 7, and 14 post-treatment, n = 3 animals (for the first cohort) or n = 2 animals (for the subsequent two cohorts) per group were sacrificed and TMs imaged.

After sacrifice, the bullae were dissected, visually inspected, and imaged. The middle ear effusion and biofilm mass were collected when present. The middle ear lavage was performed using 1ml sterile PBS. The effusion/biofilm and lavage were combined to enumerate total viable bacteria by plating and presented as CFU/ear. This study was performed with two different gel/MS instillation conditions: 1) 10mg MS/100μL gel, 100μL instilled per ear and 2) 15mg MS/100μL gel, 200μL instilled per ear.

Following sacrifice of the chinchilla cohorts treated 72h post-infection, the bullae were excised and fixed in 10% formalin. TMs were then carefully excised, dehydrated overnight in 70% ethanol, embedded in paraffin, sectioned in 5μm thick sections, and stained with hematoxylin and eosin (H&E). Paraffin histology was performed by the Tissue Culture & Histology Module within the University of Pittsburgh Department of Ophthalmology. Stained sections were imaged and evaluated using light microscopy (Leica Microsystems DM2500) with digital microscope camera (Leica Microsystems DFC295) by a blinded technician, with group/cohort masked prior to evaluation.

### 2.8. Statistical methods

Data for *in vitro* release assays, MS density, gel degradation, and cytotoxicity testing are represented as average ± standard error for at least n = 3 samples. Volume impedance measurements of MS diameter are represented as average ± standard deviation for approximately n = 15,000 particles per sample. Gel degradation data were compared using Student’s t-test at each time point. Bacterial counts from *in vivo* efficacy studies were analyzed using Kruskal-Wallis test with Dunn’s post-hoc testing to compare each time point within treatment groups. Auditory brainstem response threshold shifts from baseline were analyzed using Kruskal-Wallis test with Dunn’s post-hoc testing within each group and Mann Whitney U test for comparison between applied gel volumes. All statistical analyses were performed using GraphPad Prism software (San Diego, CA).

## 3. Results

### 3.1. Microsphere characterization

**Fig 1:**
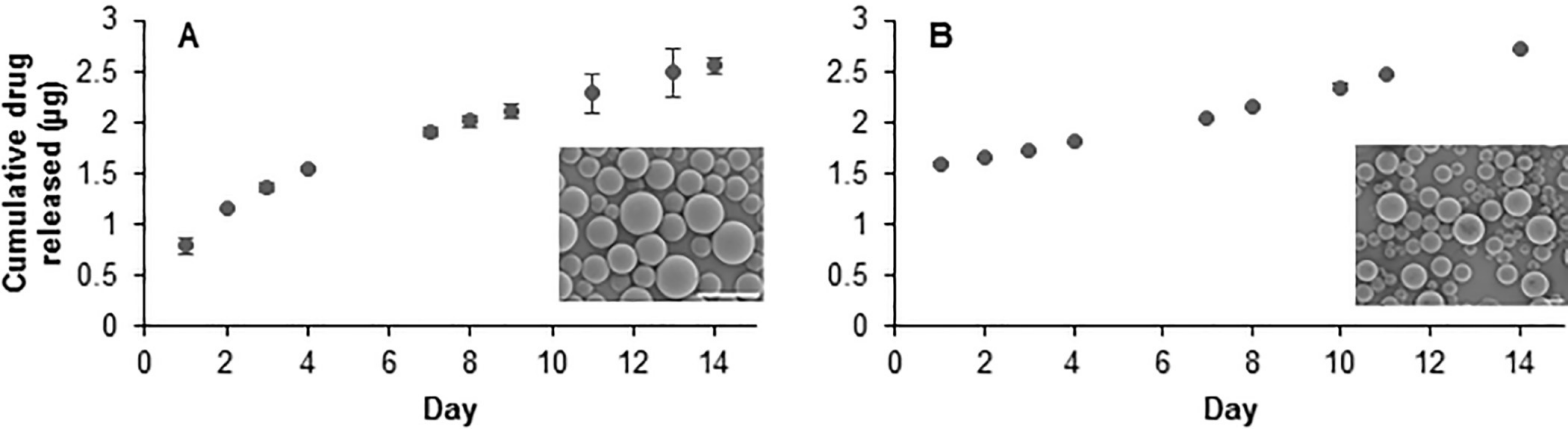
In vitro antibiotic release from microspheres. Cumulative in vitro release of (A) ciprofloxacin and (B) ceftriaxone from 10mg MS over 14 days. Error bars represent the average ± standard error for n = 3 samples. Inset: Scanning electron microscopy images of drug-loaded MS (scale bar = 10μm).

Release of ciprofloxacin follows an approximately linear pattern over 14 days (Fig 1A), while ceftriaxone-loaded MS demonstrate 58% burst release on Day 1 followed by approximately linear release over 14 days (Fig 1B). Total drug loading was taken to be cumulative release at Day 14, once all MS were degraded and release was exhausted: 2.56 ± 0.08μg ciprofloxacin released from 10mg MS and 2.71 ± 0.02μg ceftriaxone released from 10mg MS, with data shown as average ± standard error for n = 3 samples. Volume impedance measurements of microsphere diameter (Table 1) confirm visual analysis of microsphere size with scanning electron microscopy (Fig 1, inset) and are consistent with previously observed diameter ranges for similar microsphere formulations developed by this group [46,47]. Volume average diameters for blank, ciprofloxacin, and ceftriaxone MS were calculated, respectively, as: 7.85 ± 5.37, 6.88 ± 3.36, and 16.69 ± 5.65μm, with data shown as average ± standard deviation for approximately n = 15,000 samples. Ceftriaxone MS diameter was adjusted to larger diameter by decreasing homogenization speed during fabrication to reduce initial burst release and slow degradation rate to extend treatment duration [56].

**Table 1:** Average diameter and density of microspheres.

Microsphere type	Diameter (μm)	Density (mg/mL)
Blank	7.85 ± 5.37	776.9 ± 12.5
Ciprofloxacin	6.88 ± 3.36	752.5 ± 13.0
Ceftriaxone	16.69 ± 5.65	650.7 ± 14.5

### 3.2. Thermoresponsive gel characterization and retention

Swelling ratio (SR) indicates increase in gel weight due to water absorption and was determined by the following formula, where Ws refers to mass of swollen gel in water and Wd refers to dry gel:

$$Swelling\;ratio=\frac{Ws-Wd}{Wd}$$

**Fig 2:**
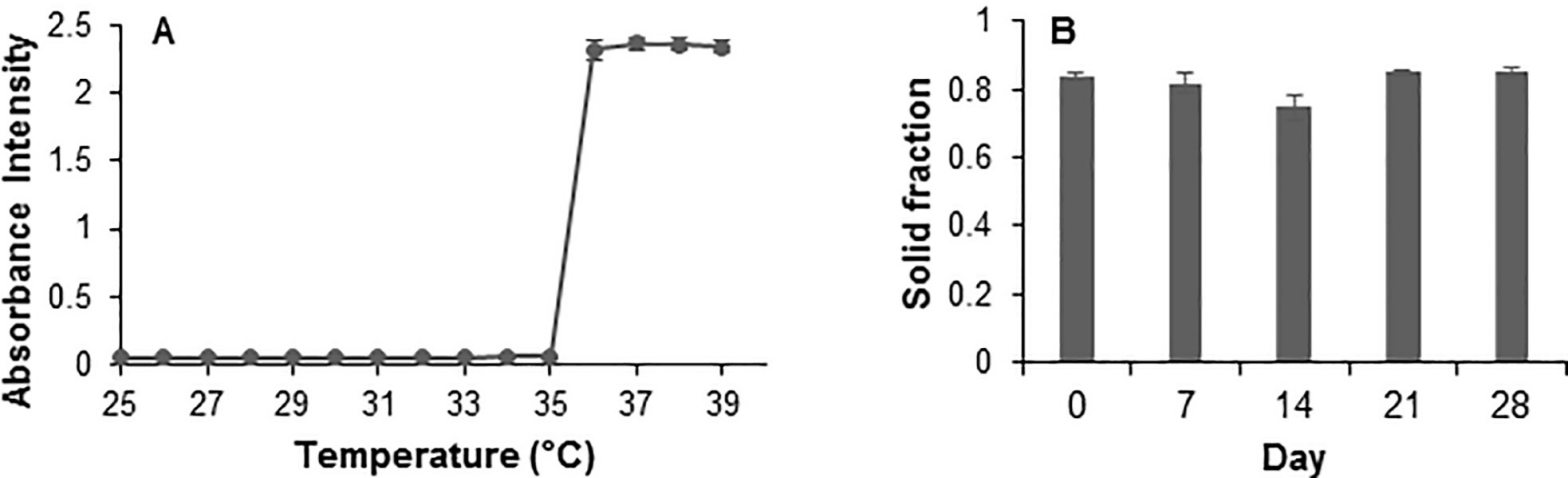
Thermoresponsive pNIPAAm gel properties. Characterization of gel properties, including (A) least critical solution temperature (LCST) determined via absorbance measurement at 415nm and (B) degradation at 37°C over 28 days. Error bars represent the average ± standard error for n = 3 gel samples. Degradation samples were analyzed for significance (p<0.05) using Student’s t-test at each time point.

Swelling ratio was calculated to be 5.13 ± 0.30 for n = 5 gel samples and is comparable to previous smaller batch results as described in Fedorchak et al 2017 [48] and other groups investigating pNIPAAM based gels [57]. LCST (Fig 2A) was determined via spectrophotometric absorbance measurements as ~35.5°C, which is also comparable to previous results and indicates gel can shed its excess water content and transition to solid form at physiological temperatures [48]. No significant change in solid fraction over 28 days (Fig 2B) indicates no degradation which is expected as pNIPAAm is non-biodegradable [50]. Solid fraction was quantified by the following formula, where *Wi* refers to mass of gel on day *i* = 7, 14, 21, 28:

$$Solid\;fraction=\frac{Wi-ave(Wd)}{Wi}$$

**Fig 3:**
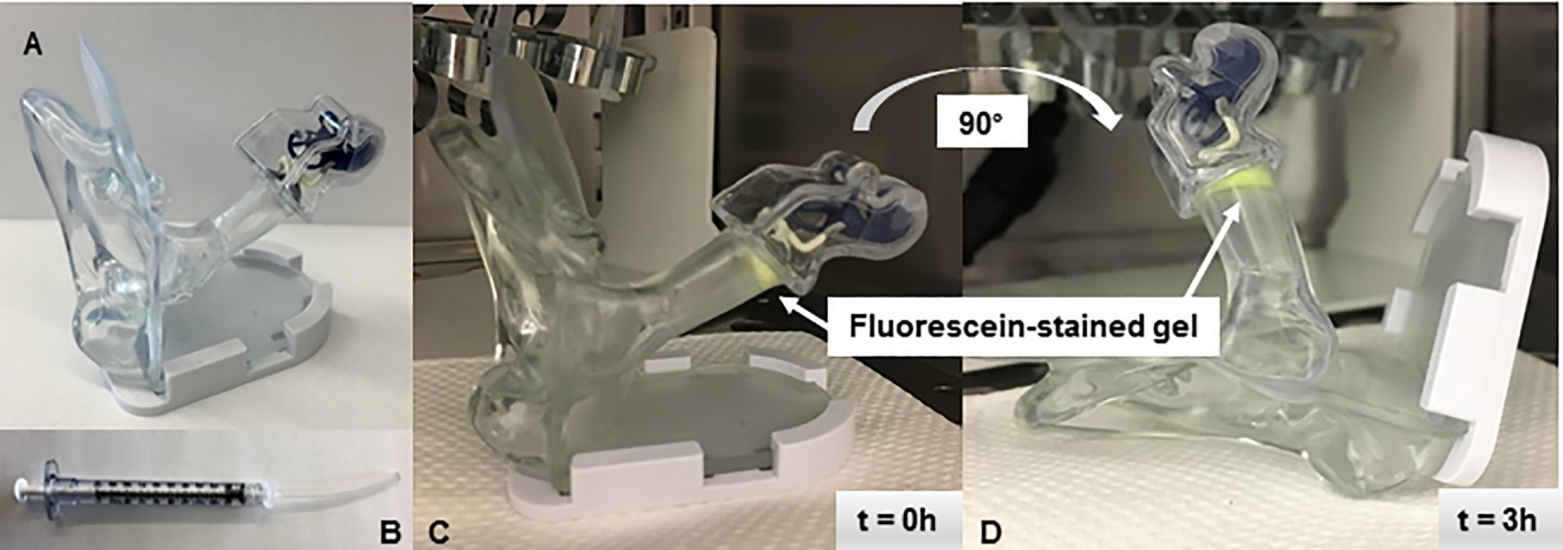
Model of drug delivery system retention. Administration of 100μL gel/MS suspension (gel stained with fluorescein for visualization) to TM in synthetic human ear model (A) using a modified syringe applicator (B). Retention was confirmed after 3 hours inversion (C,D).

Thermoresponsive gel was easily applied to the TM of an adult human ear model and retention was confirmed after 3 hours inversion at 37°C (Fig 3). The gel was left in the ear model for up to 24h, however the gel dried due to low-moisture conditions in the incubator, which should not occur *in vivo* due to humidity in the external auditory canal [58]. These results suggest that the gel drop can be instilled to the TM and retained *in situ* for the duration of treatment.

### 3.3. Cytotoxicity

**Fig 4:**
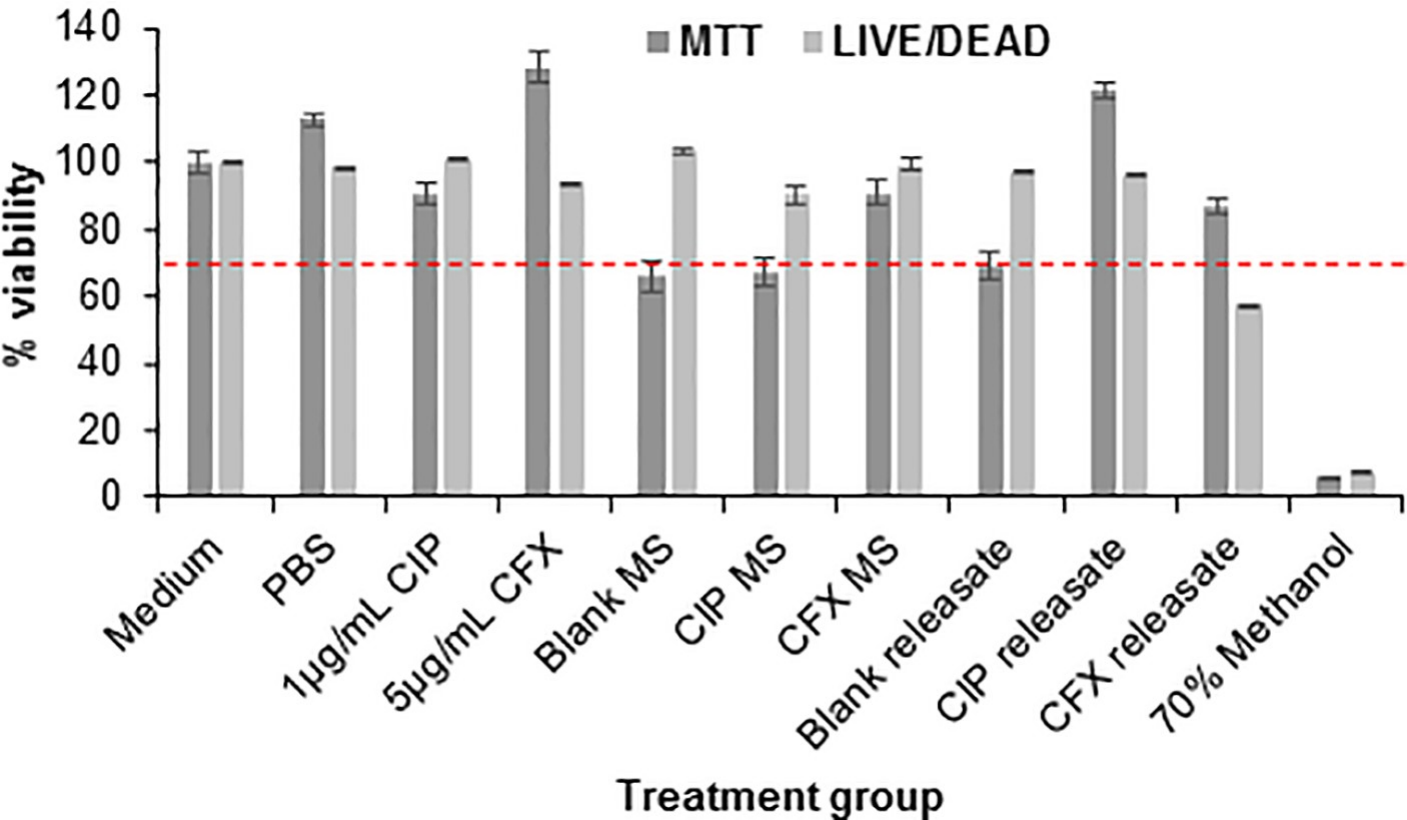
Cytotoxicity of antibiotics, microspheres, and releasates. Cell viability assays show acceptable levels of cytotoxicity due to application of blank and drug-loaded microspheres and microsphere releasates to human dermal keratinocytes for 24h. Error bars represent average ± standard error for n = 3 samples for all groups except Medium, PBS, Blank MS, and Blank releasate groups which have n = 6 samples. Red dashed line indicates 70% viability, the minimum recommended for medical devices [59–61].

Both MTT and LIVE/DEAD viability assays suggest acceptable levels of cytotoxicity following 24h of treatment with aqueous drug, blank and drug-loaded microspheres, and microsphere releasates (Fig 4). For skin-contacting medical devices, a minimum of 70% *in vitro* cytotoxicity is considered acceptable [59–61]. Aqueous ciprofloxacin and ceftriaxone resulted in, respectively, 90.4 ± 3.4% and 128.1 ± 4.6% cell viability in the MTT assay and 101.3 ± 0.1% and 94.2 ± 0.1% viability in the LIVE/DEAD assay. Blank, ciprofloxacin-loaded, and ceftriaxone-loaded MS resulted in, respectively, 66 ± 4.4%, 67.4 ± 4.1%, and 90.9 ± 3.5% viability in the MTT assay and 103.6 ± 0.9%, 90.3 ± 2.8%, and 99.3 ± 1.8% viability in the LIVE/DEAD assay. Releasates from blank, ciprofloxacin-loaded, and ceftriaxone-loaded MS resulted in, respectively, 68.8 ± 4.2, 121.8 ± 2.3%, and 86.8 ± 2.2% viability in MTT assay and 97.1 ± 0.3%, 96.5 ± 0.1%, and 57.6 ± 0.1% viability in the LIVE/DEAD assay. Positive controls of medium and PBS resulted in 100 ± 3.3% and 112.8 ± 1.8% viability in the MTT assay and 100 ± 0.5% and 98.8 ± 0.2% viability in the LIVE/DEAD assay. Negative control of 70% methanol resulted in 6.2 ± 0.2% and 7.7 ± 0.01% viability in the MTT and LIVE/DEAD assays, respectively. Results are reported as average ± standard error.

The MTT assay is a commonly used method to test medical device-related cytotoxicity; discrepancies between cell viability percentages due to use of different cytotoxicity assays have previously been noted, with the MTT assay more sensitive to detection of cytotoxicity [62,63]. These results support this observation and suggest the microspheres and releasates will be biocompatible *in vivo*. Lack of cytotoxic response due to application of gel has been previously tested and reported [48].

### 3.4. Ex vivo transtympanic permeability

**Fig 5:**
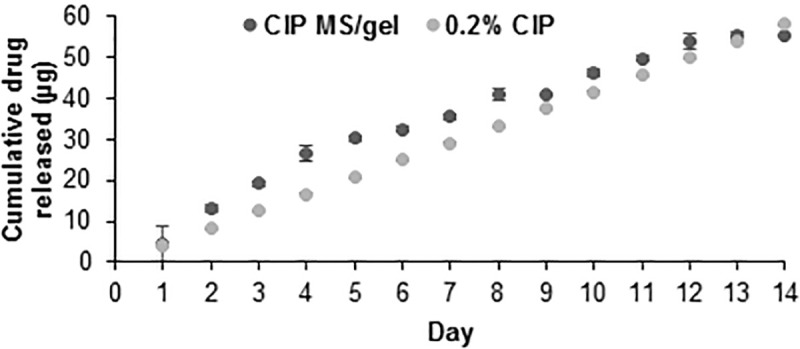
Ex vivo permeability of ciprofloxacin. Ex vivo permeability results showing transtympanic release from gel-MS system and standard twice-daily topical drops (0.2% ciprofloxacin), scaled to guinea pig ear canal volume [52] with reference to human ear canal volume [53–55]. Error bars represent the average ± standard error for n = 3 ears per time point.

*Ex vivo* studies indicate antibiotic can permeate across the tympanic membrane to the middle ear space without the use of potentially harmful chemical permeation enhancers (Fig 5). The standard topical 0.2% ciprofloxacin drops were applied in the same volume as they are typically applied in humans– 2 drops twice daily [22]. Due to size difference between guinea pig [52] and human [53–55] ears, concentration of ciprofloxacin in the receiving chamber due to treatment with these drops was scaled down by a factor of 3 to closer approximate physiological conditions. Overall volume of applied gel/MS drop can also be increased in humans as human ear canal volume is approximately 0.5–1.5mL [53–55] while 100μL gel with 10mg MS were applied in these studies due to guinea pig external auditory canal volume of approximately 200μL.

### 3.5. Bacterial killing studies

**Fig 6:**
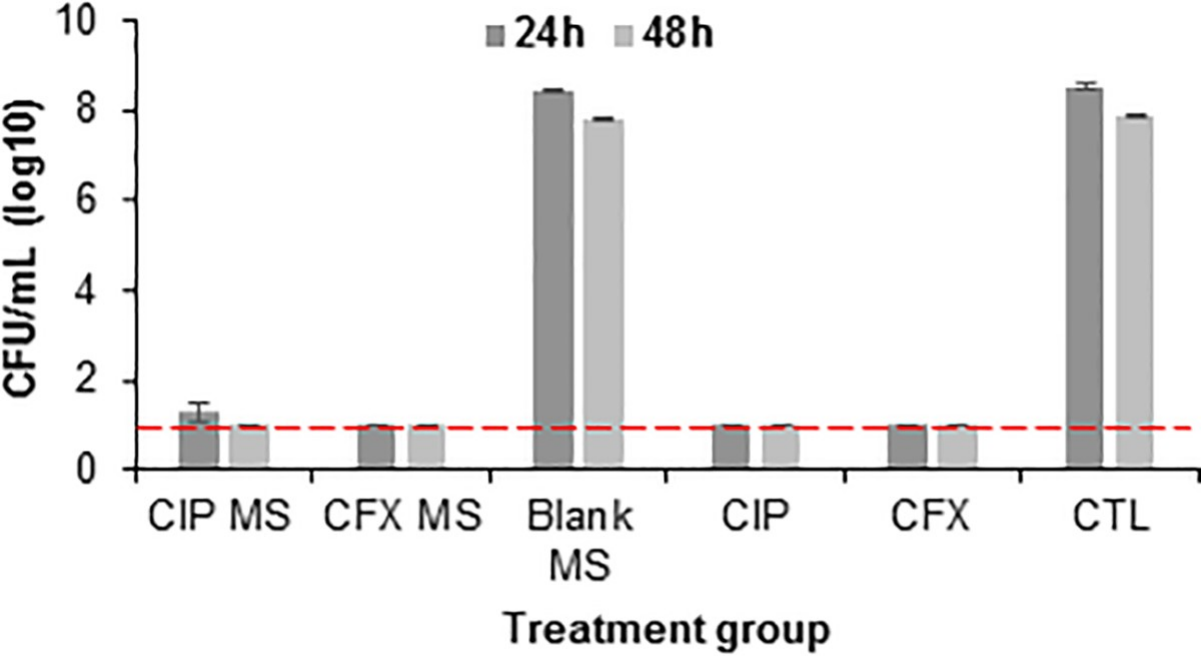
In vitro clearance of bacteria treated with antibiotics and microspheres (MS). Clearance of Haemophilus influenzae after 24-48h due to treatment with ciprofloxacin (CIP) MS, ceftriaxone (CFX) MS, blank MS, 1μg/mL CIP, 5μg/mL CFX, or no treatment control (CTL). MS treatment groups were applied in concentration of 10mg MS/mL bacterial culture. Error bars represent the average ± standard error for n = 8 samples for CIP MS and CFX MS groups and n = 4 samples for all other groups. Red dotted line indicates complete bacterial clearance.

All *H*. *influenzae* bacteria were cleared after treatment for 24h and 48h with ciprofloxacin-loaded MS, ceftriaxone-loaded MS, free ciprofloxacin, and free ceftriaxone, with significantly reduced bacterial count compared to treatment with blank (no drug loaded) MS and no treatment control (Fig 6). No significant difference between MS groups and releasate groups indicates antibiotic preserves its antibacterial capacity after encapsulation in and release from MS and is as effective at clearing bacterial infection as free antibiotic. This characterization of antibacterial efficacy allowed for justification to proceed to *in vivo* studies.

### 3.6. Auditory brainstem responses

**Fig 7:**
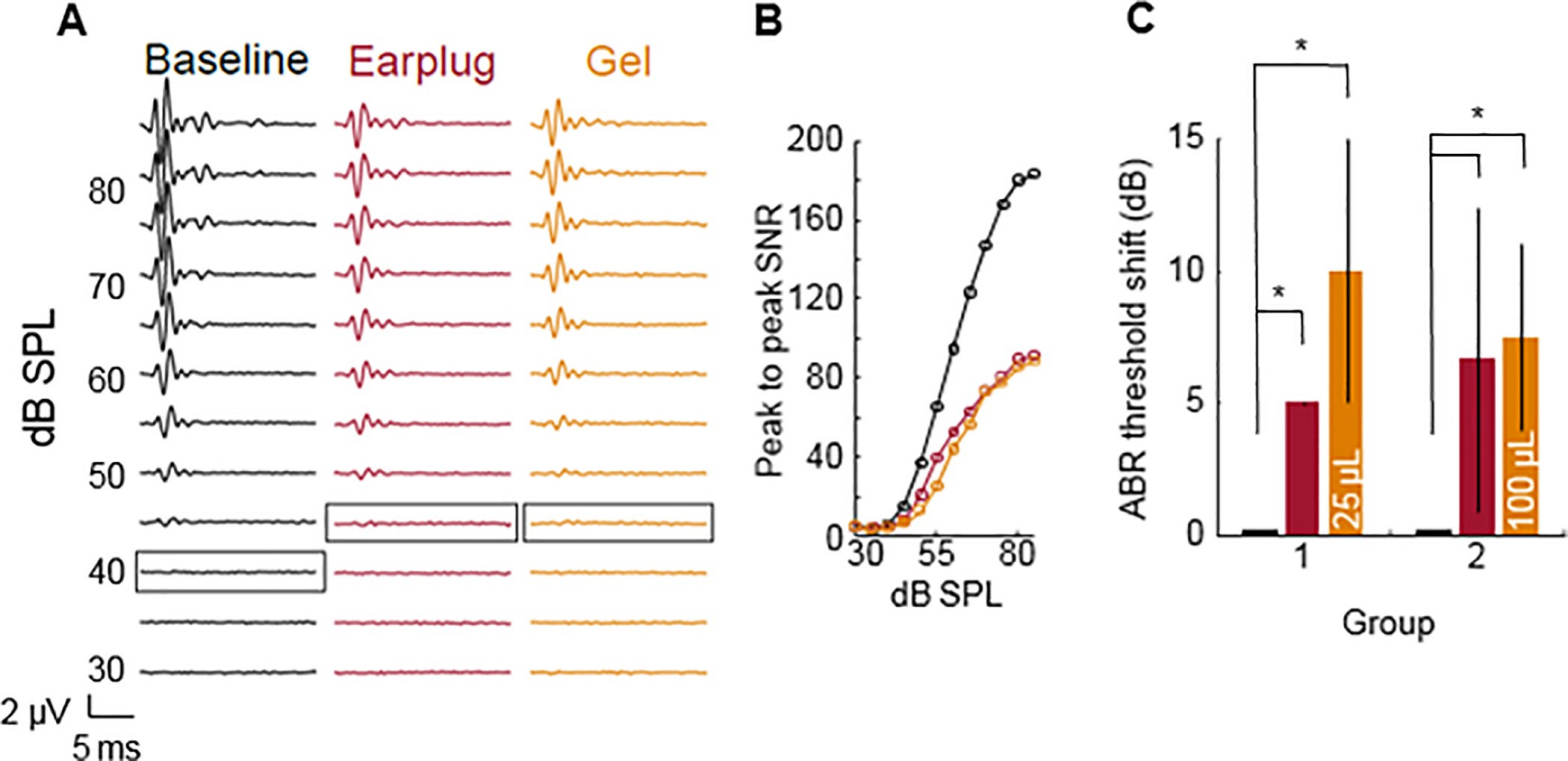
Threshold shifts recorded via auditory brainstem response (ABR) in guinea pigs. Each group was tested with both ears unplugged as baseline, left ear plugged with earplug, and right ear with gel applied. Group 1 received a 25μL gel drop and Group 2 received a 100μL gel drop. Representative ABR traces (A) and peak to peak signal to noise ratio (SNR) (B) are shown along with threshold shift relative to baseline for each group (C). Threshold shift in sound pressure level (SPL) are indicated with a box (A). Threshold shifts are represented as mean ± standard error for n = 3 animals per group (n = 2 for 100μL gel drop testing scenario). Threshold shifts within each group were analyzed for statistical significance (*p<0.05) with Kruskal-Wallis test and Dunn’s post-hoc testing. Significant differences in threshold shift due to applied gel volumes were analyzed with Mann Whitney U test.

Auditory brainstem responses (Fig 7) in two groups of guinea pigs indicate application of gel to the ear canal results in a similar threshold shift as application of an earplug. All parameters were the same for both groups except volume of gel applied. The testing scenario of 100μL contained n = 2 animals, compared to n = 3 animals in all other testing scenarios, due to difficulty of vinyl polysiloxane earplug removal causing perforation of the tympanic membrane in the left ear, confounding the gel-only ABR results for that animal. Subsequent groups consisted of a piece of foam applied to the ear prior to vinyl polysiloxane to prevent TM perforation upon removal. In both groups, there was no significant difference in threshold shift between one ear plugged with ear plug and one ear with 25μL or 100μL gel instilled. In both of these testing scenarios, the other ear was left unplugged. There was no significant difference in hearing threshold shift due to different gel volumes applied. Threshold shift of sound pressure level due to one ear containing either an earplug or gel was significantly different compared to both ears unplugged.

### 3.7. In vivo safety and efficacy studies

**Fig 8:**
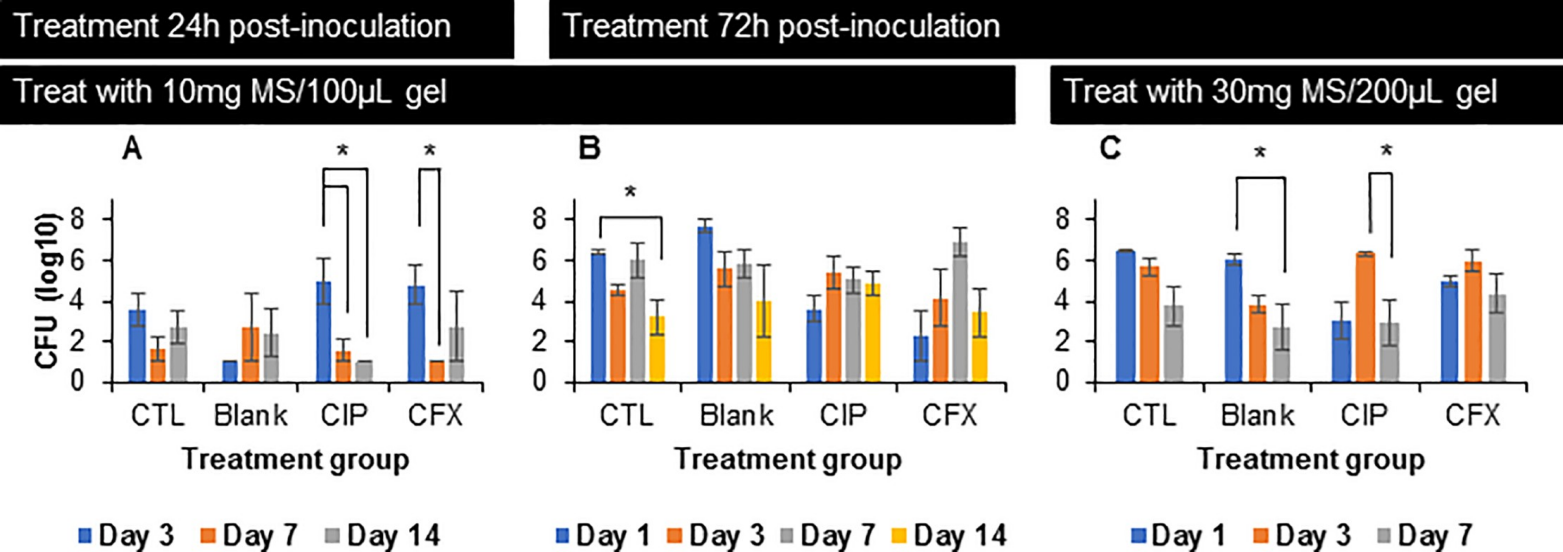
In vivo efficacy of gel/microsphere (MS) drug delivery system. Colony forming units (CFU) of *Haemophilus influenzae* bacteria in chinchilla middle ear after treatment with gel/MS containing: ciprofloxacin (CIP), ceftriaxone (CFX), blank, or no treatment control (CTL). Treatment was applied in concentrations of 10mg MS/100μL gel (A,B) or 30mg MS/200μL gel (C) at 24h (A) or 72h (B,C) after bacterial inoculation. Bacterial counts were determined upon sacrifice at time points including days 1, 3, 7, 14 post-treatment. Error bars represent the average ± standard error for n = 4 samples except groups in cohort A: CTL contains n = 9 samples and Blank, CIP, and CFX contain n = 3 samples each.

Most groups receiving no treatment (CTL) and treatment with blank MS/gel resulted in persistent bacterial infection throughout the study, confirming negative controls have no effect on bacterial clearance as expected for the disease model. For the cohort treated with 10mg MS/100μL gel 24h after inoculation with *H*. *influenzae* (Fig 8A), CIP MS/gel treatment resulted in significantly decreased bacterial count at Days 7 and 14 post-treatment, with complete clearance observed at Day 14. CFX MS/gel treatment resulted in significantly decreased infection after 7 days of treatment, with a recurrence of infection by Day 14. No significant decrease in bacteria was observed in the cohort treated with 10mg MS/100μL gel 72h after bacterial inoculation (Fig 8B). While infection persisted in all groups, the no treatment control group saw a significant decrease in bacterial infection by Day 14 post-treatment. For the subsequent cohort, treatment was increased to 30mg MS/200μL gel accordingly. In this final cohort (Fig 8C), significant decrease in bacterial infection was observed by Day 7 post-treatment in blank MS/gel group and between Day 3 and 7 in CIP MS/gel group; however, there was no significant difference between Day 1 and 7 in the CIP MS/gel group, indicating persistence of bacterial infection over treatment duration. No significant differences were observed for no treatment control or CFX MS/gel treatment. While the slight antibacterial activity seen in the blank MS group may be somewhat related to the non-zero cytotoxicity, previous studies using similar blank and antibiotic-loaded MS for treatment of endophthalmitis [47] have shown that this effect is likely not indicative of the differences seen between blank and antibiotic-loaded MS as blank MS had no effect on clearance of infection in those studies.

**Fig 9:**
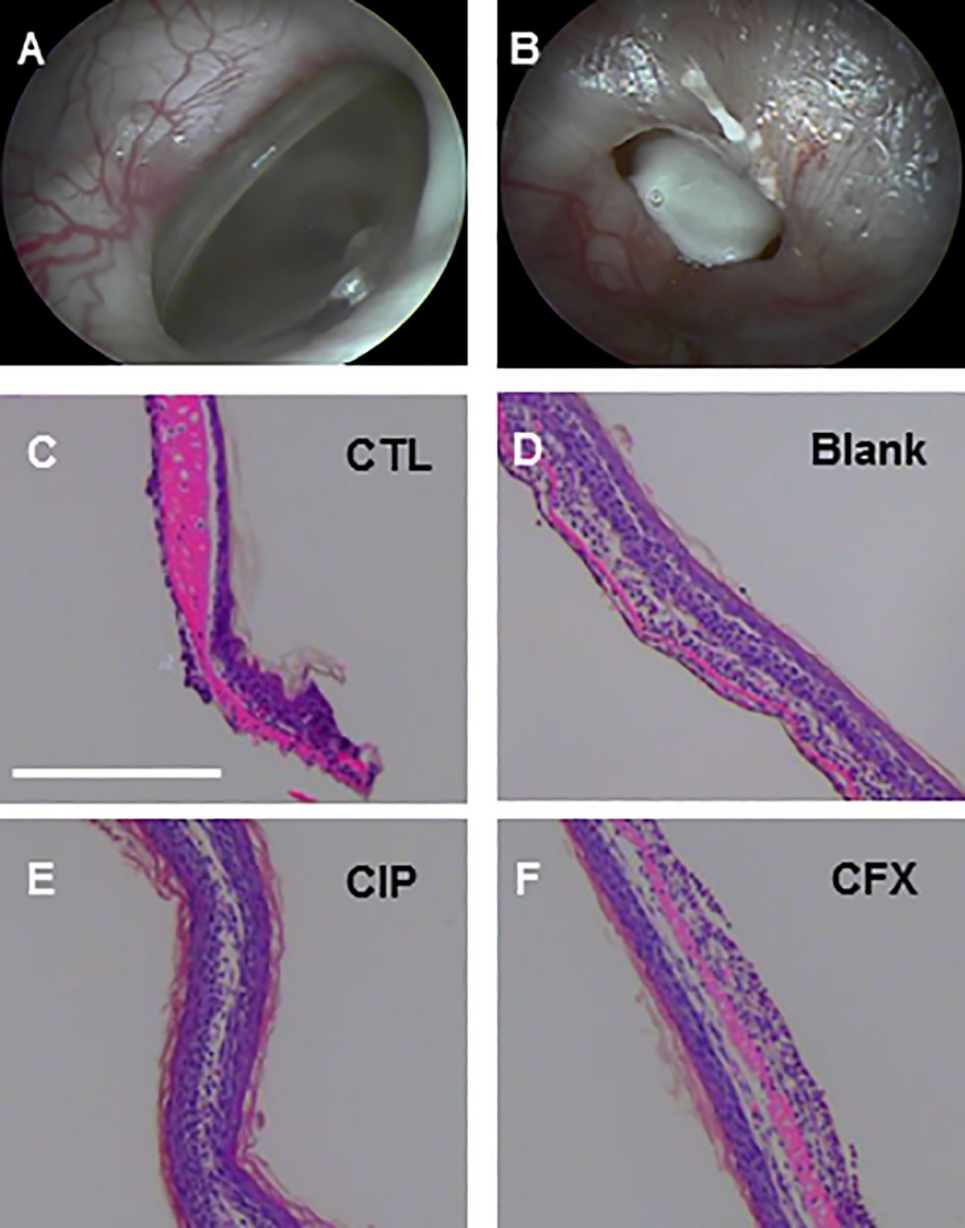
In vivo gel/microsphere (MS) placement and histopathology. Representative images of chinchilla tympanic membrane (TM) before (A) and after (B) gel/MS instillation. Representative hematoxylin and eosin (H&E)-stained sections of excised TMs representing non-treated control (CTL) and TMs treated with blank MS/gel, CIP MS/gel, and CFX MS/gel (C-F) imaged using light microscopy. Scale bar = 20μm.

H&E stained sections of excised TMs (Fig 9) indicate generally benign tissue response to treatment with MS/gel, with similar anatomy to previously reported otitis media chinchilla studies [21,32,33,64]. Significant edema is present in the interstitial layer of the control, untreated tissue (Fig 9C), as expected for inflammation inherent to OM. Enlargement in the interstitial layer is also present but less prominent in the gel/MS treated groups (Fig 9D–9F), with all tissue diameters at consistent thickness below 20μm as is expected for normal tissue [21]. Further analysis is warranted for material-specific tissue response in future studies.

## 4. Discussion

As the leading indication for antibiotics prescribed to pediatric patients in the United States, acute otitis media is responsible for over 20 million physician visits annually for the 80% of children who experience AOM [1–3]. In addition to the high incidence of physician visits, recurrent AOM can develop into chronic AOM which may require expensive and invasive surgical intervention and/or result in detrimental long-term effects on hearing, speech, and learning [7–13]. Current standards of treatment remain oral antibiotics, which come with risk of systemic side effects and rise of antibiotic resistance [3,16–18], and topical antibiotic drops, which come with issues of patient noncompliance due to frequency of dosing regimen and difficulty of administration and risk of local and systemic side effects due to high drug concentration required to overcome permeability barriers in the TM [21,25–28]. For these reasons, drug delivery systems involving thermoresponsive hydrogels with or without degradable microspheres have been investigated in previous research studies [21,29,31–33,65]. The drug delivery system developed by our group aims to improve on these previous studies by incorporating hydrolysable antibiotic-loaded polymeric MS to further control drug release into a thermoresponsive, nondegradable gel carrier that can be applied topically to the TM without need for potentially harmful chemical permeation enhancers.

*In vitro* and *ex vivo* release data indicate MS can release sufficient drug to treat the most common bacterial strains present in acute otitis media. Minimum inhibitory concentration to clear 90% (MIC_90_) of *H*. *influenzae* is 0.03μg/mL and 1–2μg/mL for *S*. *pneumonaie* [23,24,66]. Bacteria killing studies confirmed that release from antibiotic-loaded MS can kill all *H*. *influenzae* bacteria in an *in vitro* solution over 24–48 hours. A standard dose duration for AOM antibiotic prescriptions is 7–14 days [22,35,42], therefore microsphere release was tailored to last the full treatment duration. High initial antibiotic concentration followed by continuous presentation has been shown to be effective against non-responsive bacteria and biofilms, therefore this release pattern is desirable [39–41,43,67]. *Ex vivo* study of transtympanic permeability also indicates that antibiotic released from MS/gel system can permeate across the TM without the use of chemical permeation enhancers. Because this study used healthy (not infected) tissue *ex vivo*, permeability is expected to be comparable in infected ears *in vivo* and clinically due to inflammation inherent to AOM [9,68]. Transtympanic permeability of ceftriaxone due to treatment with ceftriaxone-loaded MS/gel was not tested. Due to similarity in drug release kinetics and molecular weight (MW = 554.48 and 331.35g/mol for ceftriaxone and ciprofloxacin, respectively), ceftriaxone release from MS is expected to permeate the TM as well. While these studies are not able to account for the myriad of complexities in diseased human tissues, including inflammation and increased thickness of TM and inner mucosal layer, our results suggest that sustained, localized delivery from our drug delivery system is possible.

To increase clinical translation potential, materials with a proven track record of safety were used in this study. Ciprofloxacin and ceftriaxone are currently frequently used clinically and PLGA-based materials have a strong track record of approval by the United States Food and Drug Administration [69]. *In vitro* cell viability results shown in Fig 4 confirm these findings with two different cytotoxicity assays [62,63], with minimum acceptable cell viability [59–61] achieved due to treatment with antibiotics, blank and antibiotic-loaded microspheres, and microsphere releasates. MS were applied directly to the cells in a concentration of 1mg MS/200μL media, resulting in approximately 70% cell viability after 24h. This is a high concentration of MS applied to cells, representing a worst-case scenario for cytotoxicity; however, this concentration still yields an acceptable level of *in vitro* cell toxicity with less toxicity expected *in vivo* and clinically due to less concentrated presentation and shielding by the gel carrier. Additionally, future studies can investigate optimization of washing steps during fabrication to minimize cytotoxicity, such as cell viability levels seen due to blank MS, as well as determination of best practices for sterilization of materials after fabrication.

Cell viability due to thermoresponsive gel has been previously investigated by this group, with acceptable cell viability achieved after washing steps [48]. For use in this study, gel production was scaled up tenfold and desired characteristics were maintained, as seen in Fig 3, indicating potential for larger scale production for clinical use. The gel is used as a carrier to improve retention of drug-loaded MS *in situ* and prior ocular studies by this group indicate similar efficacy *in vivo* with and without gel, but gel allows for non-invasive, simpler administration and retention [46,48]. Gel is nondegradable to improve its efficiency as a carrier for retention and to reduce potential for toxicity due to degradation products. While the gel/MS drop can be removed easily from the eye at the end of treatment duration, further investigation is necessary to determine the most effective removal system in a human ear model. Chinchilla ears are physiologically similar to human ears therefore they are a standard model for otic studies [33,65,70], however there are inherent species-specific differences in the physiology including external ear canals that are more tortuous than human external ear canals, so removal of the gel was not investigated in the chinchilla *in vivo* studies described herein.

Auditory brainstem recording studies shown in Fig 7 demonstrated no significant difference in hearing threshold shift due to the two different gel volumes applied (25μL and 100μL), indicating instilled gel/MS volume can be scaled up if needed to increase drug concentrations presented to the TM. Threshold shift of sound pressure level relative to noise floor due to one ear containing either an earplug or gel was significantly different compared to both ears unplugged. This suggests that mass effect from gel/MS treatment would have a temporary conductive effect on hearing for the treatment duration. However, there is an inherent temporary conductive hearing loss in cases of otitis media due to bulging of the inflamed tympanic membrane [15], therefore minimal attenuation of hearing during treatment duration is not expected to greatly affect patient quality of life and treatment can help prevent permanent sensorineural hearing loss, a side effect of chronic otitis media [10–13]. Further studies are needed to evaluate long term ototoxicity of the biomaterials, with an expanded look at the topical safety of ceftriaxone in particular, using longitudinal ABR testing.

While there are inherent interspecies differences in TM morphology [64], guinea pigs were used in *ex vivo* transtympanic permeability studies and chinchillas were used for *in vivo* otitis media studies. Both are standard accepted animal models for otic studies with similar TM thickness of 10μm in guinea pigs [71] and 15μm in chinchillas [64], compared to 35–150μm thickness in humans, dependent on location on the TM [9,64,71]. Guinea pigs were chosen for *ex vivo* studies due to species availability at the primary research site, the University of Pittsburgh, with *in vivo* chinchilla studies performed at the Medical College of Wisconsin using a well-established disease model for otitis media [72–74]. Further, guinea pigs are a standard model for middle and inner ear pharmacokinetics due to a large middle ear and easy access to the cochlea, while chinchillas are ideal for otitis media studies due to similar sensitivity to pathogens and disease progression at humans [65], in addition to ease of transbullar injection to inoculate middle ear with bacteria without disturbing the TM which was imperative to this study’s focus on intact TMs. *Haemophilus influenzae* was chosen as one of the leading pathogens causing AOM, accounting for approximately half of middle ear fluid isolates in children [1,72–74].

*In vivo* efficacy studies in a chinchilla model of otitis media resulted in significantly decreased infection due to treatment 24h after inoculation (Fig 8A) with CIP MS/gel, indicating ability of the drug delivery system to treat infection *in vivo* when treatment occurs at early time points after bacterial infection. We hypothesize that recurrence of infection by Day 14 after treatment with CFX MS/gel was due to the release kinetics of CFX from MS, where the burst release and subsequent linear release were effective in clearing bacteria but lower antibiotic concentrations at later time points allowed for remaining bacteria to redevelop infection. Most of the negative control groups had no effect on bacterial clearance, as expected for this disease model; however, acute otitis media in humans often clears up on its own [4], as observed by significantly decreased, but not fully cleared, bacterial infection in one no treatment group (Fig 8B) and one blank MS/gel treatment group (Fig 8C). In cohorts treated 72h after bacterial inoculation, visual observation confirmed presence of biofilm in the middle ear, potentially contributing to inability of antibiotics to clear infection [17–20]. In future work, presence of biofilm can be visualized and quantified using imaging, such as photography and confocal laser scanning microscopy. Due to this observation, the subsequent cohort was treated with increased concentration of MS in gel and increased overall volume of materials applied to 30mg MS/200μL. However, bacterial infection and biofilm formation were persistent even with this increased treatment. While the concentration of MS in the applied gel was maximized in these studies, overall volume of treatment and/or loading of drug in microspheres can be increased further to maximize permeation of antibiotic across TM, as confirmed by results in *ex vivo* transtympanic permeation (Fig 5) and ABR (Fig 7) studies. Further, high variability was observed in both control and treatment groups, as indicated by large standard deviations and recurrence of bacterial infection at later time points after decrease at earlier time points. The *in vivo* results also suggest that variability in the disease model may be decreased by separating animals into groups with or without biofilm formation for treatment with CFX or CIP, respectively. Additionally, there are some limitations to these *in vivo* studies as clinical course in humans cannot be fully extrapolated in animals due to highly variable disease development and progression in animals, with potential for the disease to not develop, to be self-limited, or to become systemic.

## 5. Conclusion

This study demonstrates that the PLGA MS/thermoresponsive gel system is capable of encapsulating and subsequently releasing up to 14 days of potentially therapeutically relevant levels of two different antibiotics for clearance of bacteria present in acute otitis media. *In vitro* cell viability and bacteria killing studies indicate these materials can be safe for *in vivo* and eventual clinical use while maintaining ability to clear bacteria. Auditory brainstem responses indicate complete coverage of TM and ability to increase volume of material applied with minimal effect on quality of life due to hearing attenuation during treatment. *In vivo* studies in a chinchilla disease model demonstrate potential for the novel drug delivery system to effectively treat bacterial infection, with future studies focusing on further microsphere optimization and improvement of the disease model, including increased antibiotic delivery and accounting for variability in the model. Histopathological evaluation indicates *in vivo* safety of the drug delivery system, with further analysis needed to determine material-specific toxicity as variability in the disease model is addressed. Future studies will also investigate incorporation of analgesic into the gel for local pain relief due to AOM [75,76,77] and encapsulation of other therapeutics into this drug delivery system for topical treatment of other otic conditions, in addition to expanded testing of long term ototoxicity.

## Supporting information

S1 Fig Ciprofloxacin standard curve determined using UV/Vis spectrophotometry. (TIF)

S2 Fig Ciprofloxacin standard curve determined using high performance liquid chromatography. (TIF)

S3 Fig Ceftriaxone standard curve determined using high performance liquid chromatography. (TIF)

S4 Fig Gel/MS drop, indicated by red arrow, viewed through reverse side of chinchilla tympanic membrane after tissue dissection post-sacrifice during *in vivo* otitis media disease model study. (TIF)
