# Supplementary figures and images for: Controlled release of ciprofloxacin and ceftriaxone from a single ototopical administration of antibiotic-loaded polymer microspheres and thermoresponsive gel
Source: PLoS One. 2020 Oct 12;15(10):e0240535. doi: 10.1371/journal.pone.0240535 (PMC7549778; doi:10.1371/journal.pone.0240535)

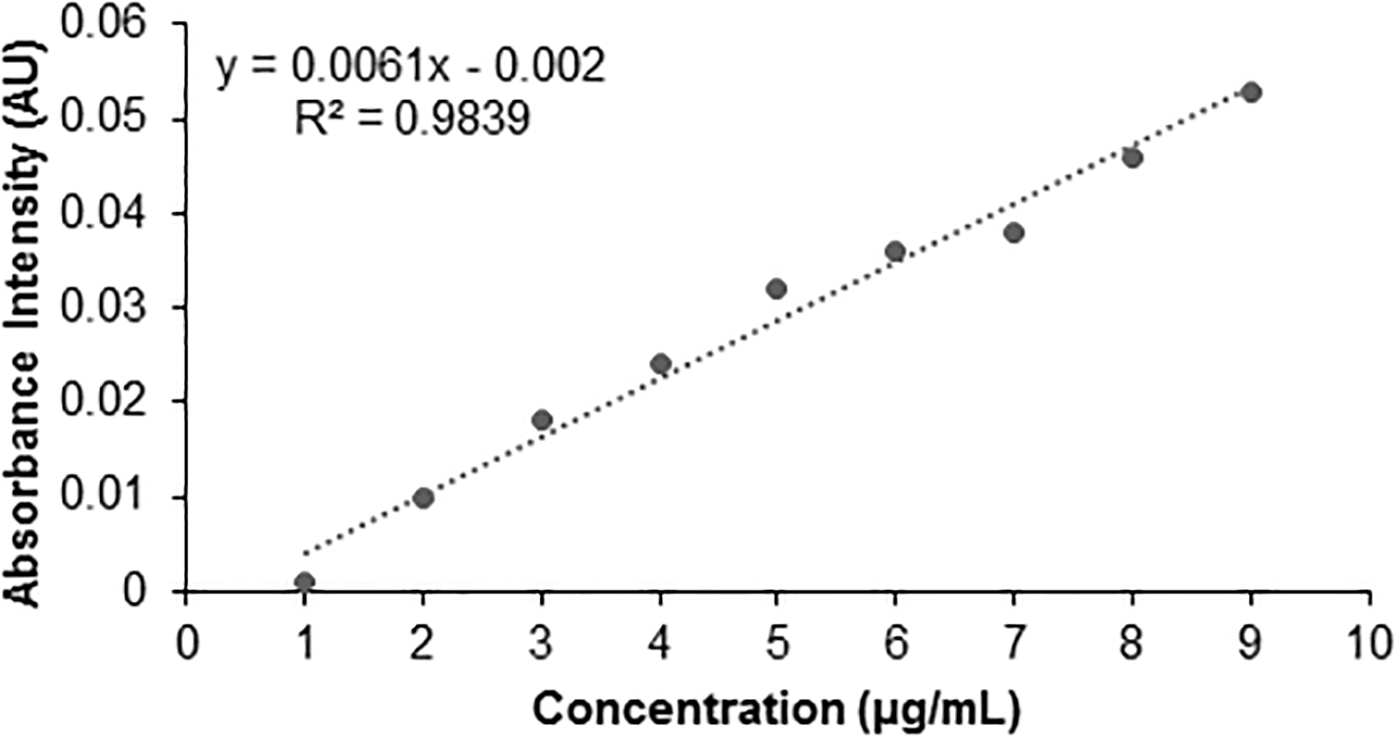

Supplement: S1 Fig — (TIF) [file pone.0240535.s001.tif]

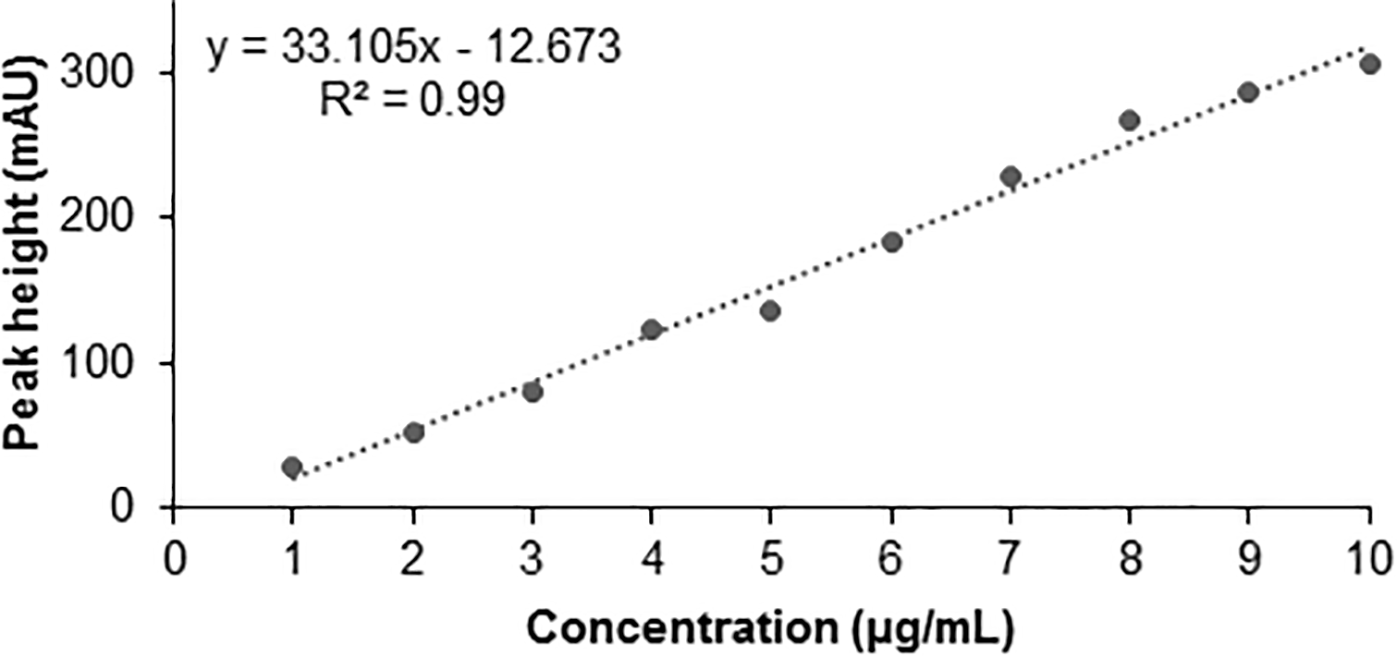

Supplement: S2 Fig — (TIF) [file pone.0240535.s002.tif]

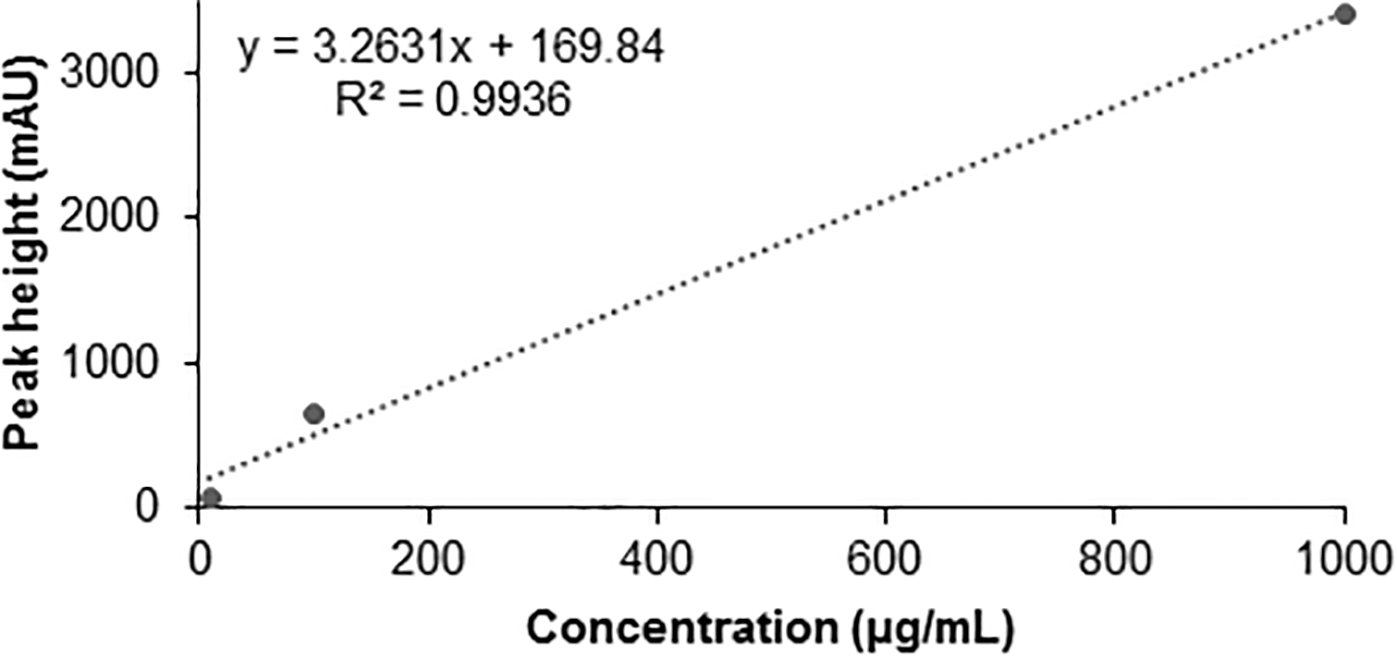

Supplement: S3 Fig — (TIF) [file pone.0240535.s003.tif]

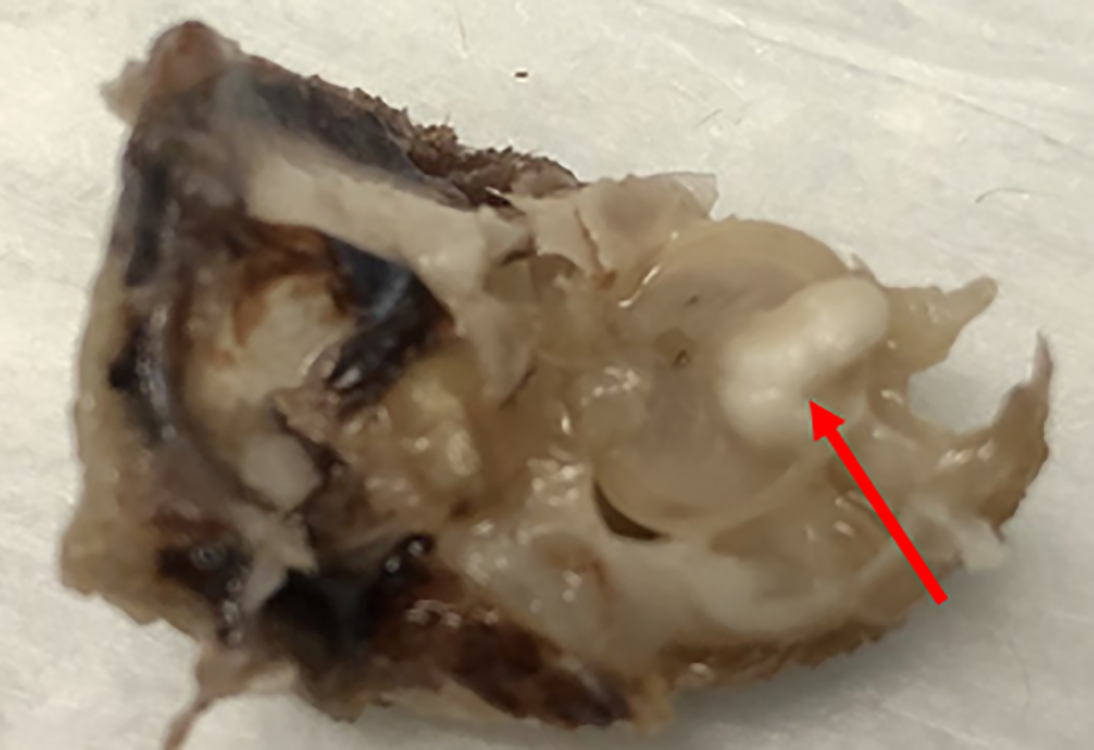

Supplement: S4 Fig — (TIF) [file pone.0240535.s004.tif]
